# Evaluating the validity and reliability of a visual dental scale for detection of periodontal disease (PD) in non-anesthetized dogs (*Canis familiaris*)

**DOI:** 10.1371/journal.pone.0203930

**Published:** 2018-09-26

**Authors:** Amy E. Bauer, Judith Stella, Matthew Lemmons, Candace C. Croney

**Affiliations:** 1 Department of Comparative Pathobiology, Purdue University, West Lafayette, Indiana, United States of America; 2 USDA-APHIS, Purdue University, West Lafayette, Indiana, United States of America; 3 MedVet, Carmel, Indiana, United States of America; Faculty of Veterinary Medicine - University of Lisbon, PORTUGAL

## Abstract

Periodontal disease is one of the most common conditions affecting companion dogs. The objectives of this cross-sectional study were: to determine 1) the agreement between visual assessments (VA) of periodontal disease (PD) performed on awake dogs and the reference standard (RS) of a dental examination and radiographs performed with the dog under general anesthesia, and 2) inter-rater reliability (IRR) for two individuals performing VAs of PD on the same dogs. One hundred and eight dogs were recruited from three veterinary practices. An oral examination and visual PD staging based on the American Veterinary Dental College’s (AVDC) 5-point scale was performed by the investigators prior to general anesthesia and the dental procedure. After the anesthetic evaluation and radiographs, the attending veterinarian staged each dog based on the AVDC’s 5-point scale. Agreement between the VA and RS as well as IRR were determined using percent agreement and a weighted kappa statistic. Eighty-nine dogs received a complete oral examination under general anesthesia with periodontal probing and full-mouth radiographs. Fifty-nine dogs received a VA by both raters. VA agreed with the RS 41.57% of the time with a weighted kappa of 0.42 (95% confidence interval 0.29–0.55), indicating moderate agreement. Between raters, VA agreed 61.02% of the time with a weighted kappa of 0.63 (95% confidence interval 0.49–0.76), indicating substantial IRR. The results of this study reinforce the idea that an oral examination in an awake dog can be a helpful screening tool, but should not be considered a comprehensive evaluation of periodontal health. In facilities housing large numbers of dogs such as shelters, breeding kennels, and research facilities, use of a VA can aid in identifying and prioritizing dogs most in need of procedures such as professional cleaning, periodontal treatment, including closed root planing, or surgical care.

## Introduction

Dental disease is consistently cited as one of the most common medical conditions of companion dogs in the United States and worldwide [1–4]. In particular, periodontal disease (PD), inflammation of the gingiva and deterioration of the bone and soft tissue structures supporting the teeth, occurs in 80% of companion dogs by 2 years of age [2]. The reported prevalence of PD and related conditions vary, possibly due to differences in the populations studied. Variation in the prevalence estimates may also be due to differences in definition of PD used in the studies. For example, in Belgium, identification of dental calculus was reported in 31% and gingivitis in 3% of dogs examined during a campaign to promote preventive medicine [5]; a prevalence of PD was reported in 60% of dogs in a Czech study [6]; and in a British study PD was reported in 9% of dogs [7].

In evaluating periodontal health, two distinct conditions can occur: gingivitis (inflammation of the gingival tissues which may or may not progress), and periodontitis in which the connective tissue support of the tooth is damaged and bone loss has occurred [8]. Evidence of bone loss is necessary to confirm periodontitis and this evidence can be collected through the use of periodontal probing and dental radiography [9, 10]. Indeed, the use of dental radiography in the assessment of dental disease has become part of the standard of dental care for companion dogs [11].

Staging the severity of PD can be challenging. In human periodontology, the first classification system was proposed by Russell in 1956. In order to facilitate epidemiologic studies of the prevalence of periodontitis, Russell proposed scoring each tooth and utilizing the mean score of all of the teeth to place the health of the mouth into 1 of 5 categories: periodontally normal, gingivitis, beginning destructive periodontitis, established destructive periodontitis, and terminal destructive periodontitis [12]. Since Russell’s initial system, several other methods of classification have been developed for use in studies of periodontitis in people [13–15].

In veterinary medicine, multiple staging techniques have also been published. A three-level categorization method was described by Colmery and Frost [16]. This categorization places the mouth into 1 of 3 categories: Group I displaying mild dental pathology (including calculus buildup); Group II with gingival recession or 4 to 6mm pocket formation; and Group III with 6 to 9mm pockets, gingival hyperplasia, and tooth mobility [16]. As the degree of PD can vary by tooth within a mouth, treatment planning has moved in the direction of evaluating each tooth as a patient within the patient [10]. A total mouth periodontal score utilizing independent scoring of each tooth for gingivitis and periodontitis has been developed [17]. Additionally, a rapid staging method has been proposed for dogs in research settings [18]. Regardless of the scale employed, the use of periodontal probing and radiographs to detect attachment loss requires anesthesia in veterinary patients. This may be problematic in facilities housing large numbers of dogs such as commercial breeding (CB) and long-term shelter (LTS) facilities where anesthetizing each dog for assessment can be both cost and time prohibitive.

A 5 stage method based on a combination of oral examination and radiographic findings has been accepted by The American Veterinary Dental College (AVDC) and is most commonly used in clinical veterinary settings at present. In this model, Stage 0 represents healthy periodontal tissues and Stage I the reversible stage of gingivitis where gums are inflamed but no bone loss has occurred [19]. While visible changes are associated with the progressive stages II-IV (mild, moderate, and severe PD), the most important defining element of these stages is bone loss [18]. This scale has been applied to dogs without anesthesia to facilitate recommendations for oral care in the clinical setting, to choose where to direct treatment resources in settings where the health of large numbers of dogs must be managed, and in studies evaluating the dental health of dogs housed in CB facilities [20]. The effectiveness of the oral examination of awake dogs in detecting PD is believed to be low, but to this point agreement between these examinations and the results of oral examinations performed under general anesthesia and including dental radiography has not been studied. The first objective of this study was to determine the agreement between visual assessments of PD performed on awake dogs (VA) and the reference standard (RS) of a dental examination, periodontal probing, and radiographs performed with the dog under general anesthesia. An additional objective was to evaluate inter-rater reliability (IRR) for 2 individuals independently performing VAs of PD on a subset of the same dogs.

## Materials and methods

### Sample size calculation

Sample size was calculated using the formula: n = n*/(1 + n*/N) where N = the population size and n* = 1/r^2^(p_a_−p_e_)^2^ where r is the relative error, p_a_ is the anticipated overall agreement probability, and p_e_ is the expected chance agreement probability [21]. As chance agreement probability was not known, p_e_ was assumed to be 0. The anticipated probability of agreement was 50%, relative error was 20%, resulting in an n* of 100. The population size was considered to be >100,000 leading to a final n of 100.

### Data collection

A convenience sample of 108 dogs presented for dental care under general anesthesia including complete oral examination and radiographs was recruited from 3 veterinary clinics that consented to participate in the study. These included one referral specialty dental practice (n = 57), one general small animal practice with a focus on veterinary dentistry (n = 29), and the community practice of a veterinary teaching hospital (n = 22). Five veterinarians performed the anesthetic procedures and radiographic assessments.

Dogs likely to show aggression were excluded from enrollment in the study. Prior to sedation, an investigator with previous experience in general veterinary practice performed a VA of each dog’s mouth. The lips and cheeks were retracted to allow the labial and buccal surfaces of the teeth and gingival margins to be examined with a focus on the gingival margins. The mouth was opened slightly to allow examination of the mandibular teeth and gingiva. A full mouth grade of PD between 0 and IV based on the tooth with the greatest level of pathologic change was recorded for each dog. The scoring system was derived from that of the AVDC [19]. As illustrated in Table 1, the scale utilized by the investigator(s) did not include the radiographic definitions for each stage of PD. For a subset of the dogs, a second investigator independently evaluated the dog’s teeth at the same visit and recorded the grade of PD in order to determine IRR. After the dental procedure was performed under general anesthesia, the attending veterinarian’s grade for PD was recorded based upon the tooth/teeth with the greatest level of pathology as detected by examination (including periodontal probing for measurement of the degree of gingival recession and attachment loss) and radiography as illustrated in Table 2. Information was also collected on the sex, neuter status, breed, and age of each dog (S1 Appendix).

**Table 1:** Rating system used for pre-anesthetic evaluation of periodontal disease.

Stage	Description
0	No plaque or calculus. Gums are normal.
I	Mild amount of plaque. Gums are mildly red
II	Moderate amount of plaque. There is redness and swelling of the gums
III	Tartar is present. Gums are receding or hyperplastic
IV	Heavy tartar is present. Gums are severely inflamed. There may be evidence of infection or tooth loss.

**Table 2:** Rating system used by attending veterinarians as reference standard.

Stage	Description
0	Clean crowns, no evidence of gingival inflammation. No bone loss on radiographs
I	Tartar buildup, gums are slightly inflamed. No bone loss on radiographs
II	Some inflammation of the gums. Bone loss of 0–25% on radiographs
III	Inflammation of the gums along with recession or hyperplasia. Bone loss of >25%
IV	Severe gingival inflammation and recession with evidence of infection. Teeth are loose and may be missing. Large degree of bone loss on radiographs

As listed in the S1 Appendix, a total of 108 dogs were initially enrolled in the study. Informed consent was obtained from the caretaker of each dog enrolled in the study. All experimental procedures were approved by the Clinical Review Panel of the Purdue University College of Veterinary Medicine and the Purdue University Animal Care and Use Committee.

Of the 108 dogs initially enrolled in the study, 11 (10.18%) did not undergo the planned anesthetic procedure, 2 (1.85%) had a diagnosis that was not PD (1 stomatitis, 1 neoplasia), and 6 (5.56%) did not have dental radiography performed. This resulted in a sample of 89 dogs for analysis of agreement with RS (Table 3). The sample included 66 purebred dogs representing 37 breeds along with 21 mixed breed dogs and 2 dogs without breed identified. Mean age was 7.85 years and ranged from 1 to 14 years. Forty-seven dogs were male (41 neutered, 6 intact), and 42 were female (39 spayed, 3 intact).

**Table 3:** Number of dogs recruited from participating veterinary practices.

Facility	Description	Number enrolled	Number included in VA-RS analysis	Number included in IRR analysis
1	Private referral specialty dental practice	57	44	29
2	Small animal general practice	29	29	18
3	Veterinary teaching hospital community practice	22	16	12

Fifty-nine dogs (including 7 dogs that did not complete the anesthetic dental procedure, 1 dog with a diagnosis of neoplasia, and 3 dogs that did not have radiographs taken and were thus not included in the RS portion of the study) were evaluated by both of the raters. The sample population consisted of 41 purebred dogs representing 31 different breeds along with 11 mixed breed dogs. Mean age was 7.56 years ranging from 1 to 14 years. Twenty-seven dogs were male (21 neutered, 6 intact) and 32 dogs were female (30 spayed, 2 intact).

### Statistical analysis

Overall percent agreement between VA and RS was calculated using the formula: % agreement = (number agreed upon/total observations) x 100%. A weighted kappa statistic was utilized to quantify agreement between the VA in the awake dog and the reference standard. For each stage identified by the RS, a percent correct was calculated as: % correct = (number correctly identified by VA/number identified by RS) x 100%. Cohen’s kappa was utilized to quantify agreement by stage. Prevalence indices (PI) were also calculated to determine the influence of homogeneity of responses on these values using the formula: PI = |s-o|/n where s indicates the number of agreed upon identifications of that stage, o indicates the number of agreed upon identifications of a different stage, and n is the total number of observations [22]. PIs can only be calculated for binary outcomes, so were not used in the assessment of overall agreement [22].

Percent agreement and a weighted kappa were also used to determine IRR between assessors in the dogs where both performed VAs. For each stage, percent agreement was calculated as: (number agreed upon/total number of times that stage was identified) x 100%. Cohen’s kappa was utilized to quantify agreement by stage and PIs were calculated as above.

Both weighted and Cohen’s kappa values were calculated using SAS, version 9.4 (SAS institute, Cary, NC, USA). Interpretation of the kappa statistic was based on the scale suggested by Landis and Koch where <0.00 indicates poor agreement, 0.00–0.20 indicates slight agreement, 0.21–0.40 indicates fair agreement, 0.41–0.60 indicates moderate agreement, 0.61–0.80 indicates substantial agreement and 0.81–1.00 indicates almost perfect agreement [23]. For the purposes of this study, the probability of Type I error (α) was defined as 0.05 and 95% confidence intervals (95% CI) were calculated in SAS for each kappa value reported.

## Results

### Agreement with the reference standard

**Fig 1:**
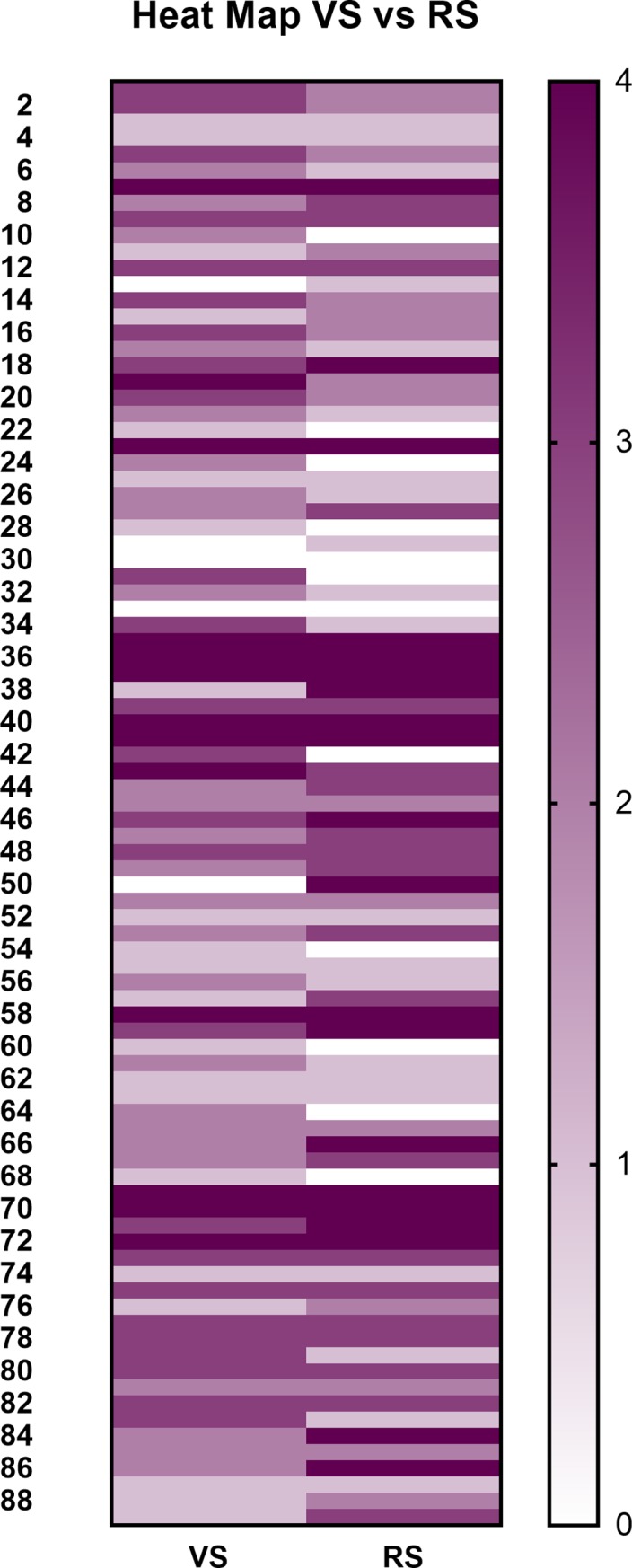
Heat map comparing the stages of periodontal disease as identified during the visual assessment and by the reference standard.

For this portion of the study it was assumed that Rater 1 and Rater 2 would have strong agreement in their visual scoring of PD. Where both raters evaluated the dog, Rater 1's score was used in analysis such that the VA score for 84 dogs was given by Rater 1 and the VA score for 5 dogs was given by Rater 2. A comparison of the distribution of PD stages as determined by the VA compared to the RS is presented in Fig 1. The overall percent agreement between the VA and the RS was 41.57% with a weighted kappa value of 0.42 (95% CI: 0.29–0.55). The measures of agreement between the VA and the RS for each stage of PD are presented in Table 4 along with the percent for each stage misclassified as either Stage 0 or Stage I. The VA agreed most strongly with the RS for detection of Stage IV PD and agreement was weakest for Stages 0 and II. Of the stages of PD where bone loss has occurred (Stage II-IV), Stage II had the highest percent misidentified as either Stage 0 or I by VA.

**Table 4:** Percent of correct identification of stage of periodontal disease by visual assessment as compared to the reference standard as well as the percent incorrectly identified as absence of disease (Stage 0) or reversible inflammation without loss of bone (Stage I).

Stage	n_VA_	n_RS_	n_agree_	% Correct	Kappa (95% CI)	PI	% 0 or I by VA
0	5	12	2	16.67	0.17 (-0.10, 0.44)	0.81	58.33 (n = 7)
I	21	21	9	42.86	0.25 (0.02, 0.48)	0.53	52.38 (n = 11)
II	25	16	5	31.25	0.03 (-0.17, 0.24)	0.54	25.00 (n = 4)
III	25	20	10	50.00	0.26 (0.04, 0.48)	0.25	10.00 (n = 2)
IV	13	20	11	55.00	0.59 (0.38, 0.81)	0.63	10.00 (n = 2)

### Inter-rater reliability

**Fig 2:**
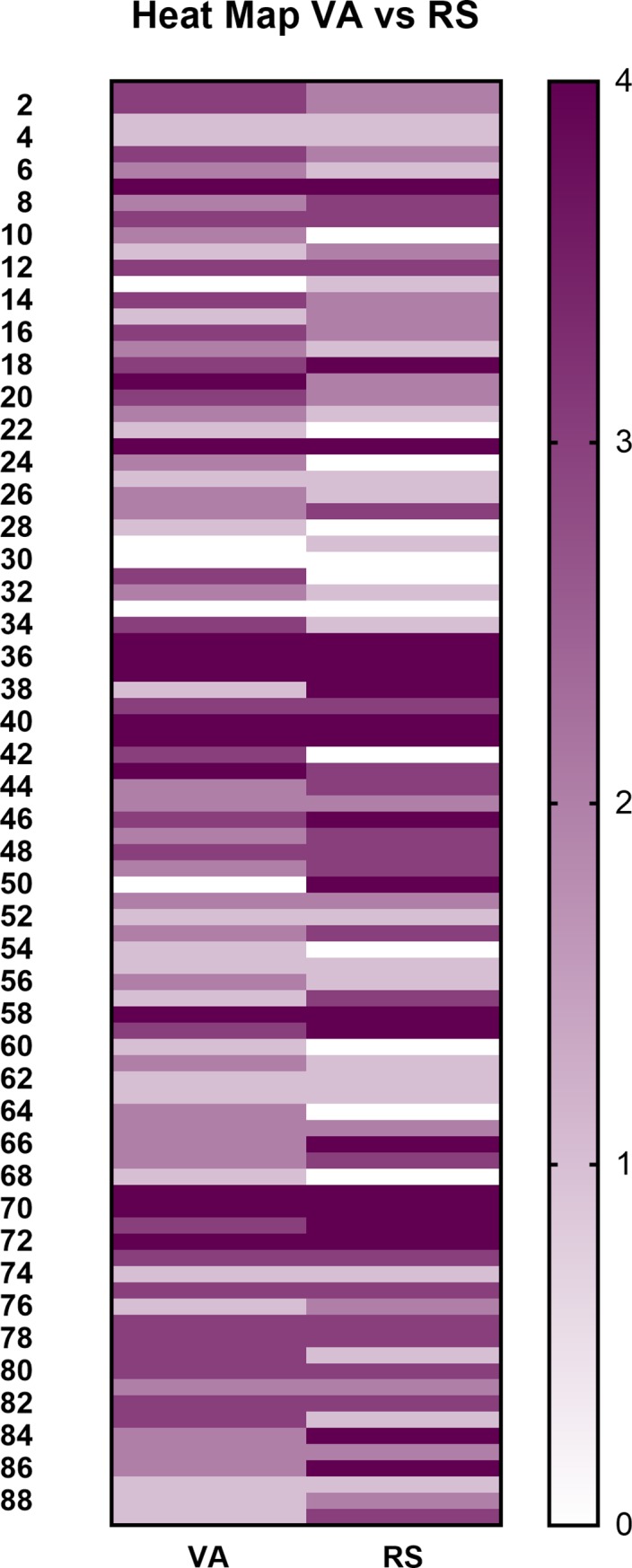
Heat map comparing the distribution of stages of periodontal disease as identified by each rater on visual assessment.

The percent distribution of stages of PD as determined by each rater is illustrated in Fig 2. The raters agreed on the stage of PD 61.02% of the time with a weighted kappa value of 0.63 (95% CI: 0.49–0.76). Agreement between the raters was strongest for Stages III and IV with an overall trend of increasing agreement with increasing severity of PD (Table 5).

**Table 5:** Percent agreement by stage between raters.

Stage	n_rater 1 only_	n_rater 2 only_	n_agree_	% Agreement	Kappa (95% CI)	PI
0	5	1	2	25.00	0.35 (-0.04, 0.74)	0.83
I	3	11	7	33.33	0.36 (0.10, 0.62)	0.52
II	8	7	10	40.00	0.39 (0.14, 0.65)	0.41
III	6	2	12	60.00	0.66 (0.44, 0.87)	0.46
IV	1	2	5	62.50	0.74 (0.46, 1.00)	0.78

## Discussion

Concern for the health, including the dental health, and welfare of dogs housed in commercial breeding kennels exists [24], but to date there has been little scientific investigation to characterize the problem. One barrier to assessment is the lack of a validated screening tool. Published screening and diagnostic methods for PD intended for use in clinical and laboratory settings [17, 18] understandably focus on procedures requiring general anesthesia as the presence of periodontitis is defined by alveolar bone loss [8]. However, there is also a need to screen for PD in field situations where anesthetic procedures are not feasible. Adapting an existing PD scale for use as a VA provides a means by which to help identify dogs at greatest need of dental care. Utilizing common metrics and terminology can aid facility managers and attending veterinarians in discussions of periodontal health and management. While there have been studies comparing methods to evaluate PD against the standard of a complete oral examination performed under general anesthesia with radiographs [18, 25, 26], the validity and reliability of VA alone have not been previously studied. The first objective of this study was to evaluate the validity of a VA to detect PD by comparing the results with a RS of a complete examination and dental radiography under general anesthesia. Moderate agreement was found, indicating that the VA has potential to aid facility managers, trained in recognizing signs of PD, to identify dogs at greatest need of professional dental care.

When agreement between the VA and RS was evaluated at the level of stage of PD, agreement was weakest for the less severe stages of PD (Stage 0, I, and II). While misclassification by the VA of RS Stages 0 and I is problematic, the direction of the misclassification is toward more severe disease, where treatment would be indicated. For example, a dog classified as Stage I on VA but Stage 0 with RS will still receive appropriate care. The weak agreement at Stage II, the stage at which irreversible bone loss may first be detected, is more concerning. In this sample, 25% of the dogs with Stage II PD by RS were incorrectly classified by VA as Stage 0 or Stage I. Depending upon the protocols at the facility, these dogs may or may not receive needed RS level care. This misclassification bias has also been noted when partial-mouth periodontal examinations (PMPE) have been used in surveillance studies of PD in people, leading to underestimation of the prevalence of PD [27]. However the use of PMPE in staging the severity of periodontal disease was not studied and thus the direction of the misclassification (more versus less severe) cannot be determined. Nevertheless, because of this risk of underestimation, attending veterinarians and facility managers at CB and LTS facilities utilizing VA should consider including management strategies for dogs with VA scores of Stage I and Stage II PD in their plans of veterinary care [28].

The second objective of the study was to determine IRR of the VA. In this study, IRR was substantial, which means that this assessment of PD may be an effective part of routine health monitoring within a facility even when different caretakers perform the VA. While it is important to note that both of the investigators performing VAs had past experience in general veterinary practice, with proper training lay assessors should also be able to achieve high levels of agreement. Future studies should include the development of training materials geared toward caretakers responsible for monitoring oral health at CB and LTS facilities and evaluation of IRR both before and after training.

When IRR was evaluated at the level of stage of PD, it followed the same pattern as the comparison between VA and RS, weakest for Stages 0, I, and II. This indicates that additional diagnostic tools for use in unanesthetized dogs may be helpful in screening and designing treatment plans at CB or LTS facilities. Biomarkers that have been investigated as aides to staging periodontal disease include measurements of serum ionized calcium [25, 29] and dissolved thiol, a product of bacterial metabolism that can indicate active periodontal infection [26]. These tools have been evaluated for use in differentiating between dogs with gingival inflammation alone as compared to those with attachment loss. Evaluating serum ionized calcium requires venipuncture and unless included with routine hematologic testing, may not be practical in CB and LTS facilities. Currently, the only biomarker testing kit commercially available for use in dogs is a product that tests for the presence of dissolved thiol at the gingival margin. In a clinical setting, use of this product was found to increase compliance with dental recommendations [30]. Use of this product in conjunction with a VA could provide caretakers working with large numbers of dogs valuable information for treatment and financial planning, particularly in dogs with VA scores at Stage I or II. Additional studies are needed to determine how measurements of dissolved thiol may be most effectively added to a veterinary care plan at CB and LTS facilities.

This study does have limitations. We anticipated that recruitment of dogs already scheduled for dental care would lead to bias in the direction of more severe disease (Stages III and IV). While 45% of the dogs enrolled in the study were diagnosed with Stage III or IV PD, an additional 37% presented with Stage 0 or the reversible Stage I, resulting in a more balanced sample than anticipated. Collecting data from different practices, with different veterinarians assessing radiographs also introduces bias, related to the element of subjectivity in radiographic interpretation. Studies of agreement between veterinary radiologists and between endodontists and a radiologist demonstrated agreement ranging from fair to substantial depending upon the pathology being assessed [31, 32], indicating that even among specialists interpretation of oral radiographs retains an element of subjectivity. In order to account for the effect of this subjectivity, future studies should incorporate measures to evaluate agreement between the clinicians evaluating the dental radiographs. Older dogs and smaller breeds have been found to be more likely to have PD. Knowledge of these factors may have predisposed the raters to identify a more severe level of PD in older or smaller breed dogs. Future studies may focus on identifying whether or not the age or breed of a dog affects the rater’s evaluation. Finally, we anticipated that all dogs recruited would complete a dental procedure with radiographs under general anesthesia but this did not occur. Due to this smaller sample size, the actual level of agreement between VA and RS may differ from that presented in this study so that generalizations should be made with care. While 95% confidence intervals indicate that the smaller sample size did not overly influence the overall agreement and IRR statistics in this study, the loss of study subjects may illustrate the importance of both cost and client understanding of the necessity of treatment in compliance with recommendations for care [33, 34]. Just as these factors are important for caretakers of companion dogs, they are likely also critical components of treatment planning among managers of CB and LTS facilities. Additional research is needed to identify which factors are most critical to address in order to improve dental care for dogs housed in CB and LTS facilities.

The AAHA-AVMA Canine Preventive Healthcare Guidelines state that a comprehensive physical examination including dental assessment should be performed at least annually [35]. The Guidelines for Standards of Care in Animal Shelters state that apparently healthy animals should be examined by a veterinarian twice annually [36]. Facility managers may find a need to perform assessments more frequently depending upon the characteristics of the dogs in their care, including breed and age. Important oral pathologies can be detected with an examination performed on a cooperative awake patient, but more subtle pathologies, including those occurring beneath the gingival margin, may be missed. This study reinforces that an oral examination in an awake dog can be a useful screening tool for PD in facilities housing large numbers of dogs, where a comprehensive evaluation under general anesthesia is not feasible for every dog. Proper use of a VA as a screening tool can help to identify the dogs at greatest need of dental care, including the general anesthesia, oral examination, and dental radiography needed for definitive detection of early alveolar bone loss and therapeutic planning.

## Supporting information

S1 Appendix Demographic and periodontal evaluation data table. (XLSX)
